# Predictors of morbidity and oncological benefit after liver resection with vascular reconstruction: international collaborative study from expert centres

**DOI:** 10.1093/bjsopen/zrag085

**Published:** 2026-09-28

**Authors:** Victor Lopez-Lopez, Francesca Ratti, Cecilia Maina, Juan Gonzalez-Ferez, Sarah Daniels, Nikhil Suresh, Safi Dokmak, Elias Karam, Augustin Suffisseu, Yawen Dong, Zhihao Li, Rory L Smoot, Muhammad Ahmad Nadeem, Federico Aucejo, Emilio Vicente, Enrico Gringeri, Eleonora Nieddu, Jordi Navinés, Lucia Hernandez, Mohammad H Fard-Aghaie, Jens Rolinger, Leyre Serrablo, Sanjay Govil, William Archie, Dionisios Vrochides, Martin de Santibañes, Eduardo de Santibañes, Fabrizio Di Benedetto, Silvio M P Balzan, Cristina Dopazo, Vicente Borrego Estella, Giuseppe Kito Fusai, Fernando Rotellar Sastre, Roberto I Troisi, Alejandro Serrablo, Silvio Nadalin, Karl J Oldhafer, Esteban Cugat, Umberto Cillo, Andrea Schlegel, Patrick Starlinger, Mickael Lesurtel, Luca Aldrighetti, Pierre Alain Clavien, Daniel Azoulay, Peter Lodge, Ricardo Robles Campos

**Affiliations:** Department of Surgery and Transplantation, Virgen de la Arrixaca Clinic and University Hospital, IMIB-Pascual Parrilla, Murcia, Spain; Hepatobiliary Surgery Division, IRCCS San Raffaele Scientific Institute, Milan, Italy; Vita Salute San Raffaele University, Milan, Italy; Hepatobiliary Surgery Division, IRCCS San Raffaele Scientific Institute, Milan, Italy; Vita Salute San Raffaele University, Milan, Italy; Group of Applied Mathematics in Science and Engineering, Faculty of Computer Science, University of Murcia, Murcia, Spain; Hepato-Bilio-Pancreatic and Transplant Unit, St James’s University Hospital, Leeds Teaching Hospitals NHS Trust, Leeds, UK; Hepato-Bilio-Pancreatic and Transplant Unit, St James’s University Hospital, Leeds Teaching Hospitals NHS Trust, Leeds, UK; Department of Hepato-Bilio-Pancreatic Surgery and Liver Transplantation, Beaujon Hospital, Assistance Publique—Hôpitaux de Paris, Clichy, France; University Paris Cité, Paris, France; Centre de Recherche sur l’nflammation, INSERM Unité 1149, Clichy, France; Department of Hepato-Bilio-Pancreatic Surgery and Liver Transplantation, Beaujon Hospital, Assistance Publique—Hôpitaux de Paris, Clichy, France; University Paris Cité, Paris, France; Department of Hepato-Bilio-Pancreatic Surgery and Liver Transplantation, Beaujon Hospital, Assistance Publique—Hôpitaux de Paris, Clichy, France; Department of Surgery, Division of Hepatobiliary and Pancreas Surgery, Mayo Clinic, Rochester, Minnesota, USA; William J. von Liebig Center for Transplantation and Clinical Regeneration, Mayo Clinic, Rochester, Minnesota, USA; Department of Surgery, Division of Hepatobiliary and Pancreas Surgery, Mayo Clinic, Rochester, Minnesota, USA; Department of Inflammation and Immunity, Lerner Research Institute, Cleveland Clinic, Cleveland, Ohio, USA; Transplantation Center, Digestive Disease and Surgery Institute, Cleveland Clinic, Cleveland, Ohio, USA; Department of Inflammation and Immunity, Lerner Research Institute, Cleveland Clinic, Cleveland, Ohio, USA; Transplantation Center, Digestive Disease and Surgery Institute, Cleveland Clinic, Cleveland, Ohio, USA; General Surgery Department, Sanchinarro University Hospital, Instituto de Investigación Sanitaria HM Hospitales, Jose Camilo Cela University, Madrid, Spain; Hepato-Bilio-Pancreatic Surgery and Liver Transplantation Unit, Padova University Hospital, Padova, Italy; Hepato-Bilio-Pancreatic Surgery and Liver Transplantation Unit, Padova University Hospital, Padova, Italy; Department of General and Digestive Surgery, Hospital Germans Trias i Pujol, Barcelona, Spain; Department of Surgery and Transplantation, Virgen de la Arrixaca Clinic and University Hospital, IMIB-Pascual Parrilla, Murcia, Spain; Asklepios Campus Hamburg, Department of Surgery, Division of Hepato-Bilio-Pancreatic-Surgery, Asklepios Hospital Barmbek, Semmelweis University Budapest, Hamburg, Germany; Department of General, Visceral, and Transplant Surgery, University Hospital Tuebingen, Tuebingen, Germany; Hepato-Bilio-Pancreatic Surgical Division, Miguel Servet University Hospital, Zaragoza, Spain; Department of Hepatobiliary Surgery and Liver Transplantation, Apollo Hospital, Karnataka, India; Division of Abdominal Transplantation, Carolinas Medical Center, Atrium Health, Wake Forest University School of Medicine, Charlotte, North Carolina, USA; Division of Abdominal Transplantation, Carolinas Medical Center, Atrium Health, Wake Forest University School of Medicine, Charlotte, North Carolina, USA; Department of General Surgery, Hepato-Pancreato-Biliary and Liver Transplant Unit, Hospital Italiano de Buenos Aires, Buenos Aires, Argentina; Department of General Surgery, Hepato-Pancreato-Biliary and Liver Transplant Unit, Hospital Italiano de Buenos Aires, Buenos Aires, Argentina; Hepato-Pancreato-Biliary Surgery and Liver Transplantation Unit, University Hospital of Modena ‘Policlinico’, University of Modena and Reggio Emilia, Modena, Italy; Department of Surgery, Hospital Moinhos de Vento, Porto Alegre, Brazil; Hepatobiliary and Pancreatic Surgery Center, Saint Gallen Clinic, Santa Cruz do Sul, Brazil; Department of Hepatobiliary Surgery and Liver Transplantation, Vall d'Hebron University Hospital, Autonomous University of Barcelona, Barcelona, Spain; General and Digestive Surgery Department, HCU Lozano Blesa of Zaragoza, Hospital Clínico Universitario Lozano Blesa, Saragossa, Spain; Hepato-Bilio-Pancreatic and Liver Transplant Unit, Royal Free Hospital, London, UK; Department of General Surgery, Clínica Universidad de Navarra, University of Navarra, Pamplona, Spain; Department of Clinical Medicine and Surgery, Division of Hepato-Bilio-Pancreatic, Minimally Invasive and Robotic Surgery, Transplantation Center, Federico II University Hospital, Naples, Italy; Hepato-Bilio-Pancreatic Surgical Division, Miguel Servet University Hospital, Zaragoza, Spain; Department of General, Visceral, and Transplant Surgery, University Hospital Tuebingen, Tuebingen, Germany; Asklepios Campus Hamburg, Department of Surgery, Division of Hepato-Bilio-Pancreatic-Surgery, Asklepios Hospital Barmbek, Semmelweis University Budapest, Hamburg, Germany; Department of General and Digestive Surgery, Hospital Germans Trias i Pujol, Barcelona, Spain; Hepato-Bilio-Pancreatic Surgery and Liver Transplantation Unit, Padova University Hospital, Padova, Italy; Department of Inflammation and Immunity, Lerner Research Institute, Cleveland Clinic, Cleveland, Ohio, USA; Transplantation Center, Digestive Disease and Surgery Institute, Cleveland Clinic, Cleveland, Ohio, USA; Department of Surgery, Division of Hepatobiliary and Pancreas Surgery, Mayo Clinic, Rochester, Minnesota, USA; Department of Hepato-Bilio-Pancreatic Surgery and Liver Transplantation, Beaujon Hospital, Assistance Publique—Hôpitaux de Paris, Clichy, France; University Paris Cité, Paris, France; Hepatobiliary Surgery Division, IRCCS San Raffaele Scientific Institute, Milan, Italy; Vita Salute San Raffaele University, Milan, Italy; Department of Surgery and Transplantation, University of Zurich, Zurich, Switzerland; Centre Hépato-Biliaire, Department of Surgery, Hôpital Paul Brousse, Assistance Publique-Hôpitaux de Paris, Villejuif, France; Hepato-Bilio-Pancreatic and Transplant Unit, St James’s University Hospital, Leeds Teaching Hospitals NHS Trust, Leeds, UK; Department of Surgery and Transplantation, Virgen de la Arrixaca Clinic and University Hospital, IMIB-Pascual Parrilla, Murcia, Spain

**Keywords:** tertiary referral centres, advanced hepatobiliary surgery, surgical outcomes

## Abstract

**Background:**

Liver resection with vascular reconstruction is a highly complex procedure for advanced hepatobiliary tumours. This study evaluated the postoperative and oncological outcomes of this patient cohort.

**Methods:**

This retrospective multicentre study included patients undergoing liver resection with vascular reconstruction between 1990 and 2024 across 21 expert centres. Reconstructions involved the inferior vena cava, hepatic veins, portal vein, or hepatic artery. Primary endpoints were postoperative morbidity and 90-day mortality. Secondary endpoints included overall survival (OS), disease-free survival (DFS), and predictors of complications. Multivariable logistic regression, Kaplan–Meier, and Fine–Gray analyses were performed.

**Results:**

In all, 532 patients were included and classified according to surgical approach as *in situ* (455, 85.3%), *ante situm* (48, 9.2%), or *ex situ* (29, 5.5%). The most frequent indications were colorectal liver metastases (162, 30.4%), intrahepatic cholangiocarcinoma (139, 26.1%), and perihilar cholangiocarcinoma (106, 19.9%). The median operative time was 390 minutes. Clavien-Dindo ≥ IIIa occurred in 217 patients (40.8%), and the 90-day mortality rate was 9.8% (52). Portal vein reconstruction independently predicted post-hepatectomy liver failure, ascites, infectious complications, and major morbidity, whereas inferior vena cava reconstruction showed a more favourable perioperative profile. Hepatic vein reconstruction was associated with greater transfusion requirements and longer operative time. Vascular graft-related complications occurred in 22.3% (58) of reconstructions and were not associated with graft type. After excluding patients with perihilar cholangiocarcinoma (426), *ante situm* and *ex situ* procedures were associated with higher rates of Clavien-Dindo ≥ IIIa than *in situ* procedures (67.3% (33) and 64.3% (18) *versus* 35.5% (124), respectively; *P* < 0.001), longer intensive care unit stay (6.5 and 5 *versus* 3 days, respectively; *P* < 0.001), and higher mortality (16.3% (8) and 17.9% (5) *versus* 8.3% (29), respectively; *P* = 0.060). DFS was superior for hepatocellular carcinoma (*P* = 0.011), whereas perihilar cholangiocarcinoma showed a higher risk of recurrence (*P* = 0.022). Five-year OS was highest for sarcoma (68.8%) and hepatocellular carcinoma (42.7%).

**Conclusion:**

Liver resection with vascular reconstruction is feasible in specialized centres but remains associated with substantial morbidity and mortality. The *in situ* approach showed the most favourable perioperative outcomes, although oncological benefit varied according to tumour biology, highlighting the importance of careful patient selection.

## Teaser

This international multicentre study of 532 patients undergoing liver resection with vascular reconstruction demonstrates that extreme liver surgery is feasible in expert centres but remains associated with substantial morbidity (40.6% major complications) and 90-day mortality (9.6%). The *in situ* approach was associated with lower morbidity and resource utilization than *ante situm* and *ex situ* techniques, whereas portal vein reconstruction independently predicted worse postoperative outcomes. Long-term survival varied according to tumour biology, highlighting the importance of careful patient selection and the evolving role of transplant oncology.

## Introduction

Hepatectomies requiring vascular reconstruction represent one of the greatest challenges in modern hepatobiliary surgery due to their high technical complexity and associated risks. These interventions are indicated in patients with advanced primary liver tumours or liver metastases involving critical vascular structures, offering, in some cases, the only curative-intent therapeutic option^1^. However, the need to preserve an adequate volume of functional liver parenchyma, along with prolonged ischaemia times and the inherent complexity of vascular reconstruction, entails a high risk of postoperative complications, including liver failure, haemorrhage, infections, and vascular graft thrombosis^2^. These factors ultimately determine postoperative mortality, which has historically been reported as prohibitively high.

In recent decades, advances in surgical techniques and perioperative management have significantly improved the outcomes of these procedures. Techniques such as *ex situ*, *ante situm*, and *in situ* approaches have been implemented in specialized centres with variable results^3–6^. Similarly, the integration of advanced strategies such as hepatic perfusion and the use of extracorporeal bypass have been adopted by different centres, with potential benefits in reducing postoperative complications and protecting liver parenchyma during prolonged ischaemia periods^7–10^. Nevertheless, the implementation of these strategies remains non-standardized, resulting in variability in outcomes and hindering objective comparison between surgical approaches.

Another major challenge in this context is the selection of candidates for these procedures. Currently, there are no universally accepted criteria to accurately determine which patients would benefit most from extreme liver surgery with vascular reconstruction. This has led to decision-making being largely based on the experience of the surgical team and the resources available at each centre. The lack of studies with significant case series and homogeneous data on long-term oncological and functional outcomes hamper the optimization of surgical strategies and the development of clear guidelines for patient selection.

In this context, there is a growing need for a multicentre analysis to consolidate knowledge in this field of liver surgery and to establish clinical guidelines based on robust evidence. Standardization of surgical techniques and perioperative strategies could help reduce complications and improve oncological outcomes, as well as strengthen the capacity of surgical teams to perform these procedures in high-complexity settings.

## Methods

### Study design

This was a retrospective international multicentre study that included patients from 21 hospitals worldwide who underwent hepatectomy with vascular reconstruction due to primary or secondary hepatobiliary tumours between January 1990 and June 2024. The institutional review board at the Clinic and University Virgen de la Arrixaca Hospital (Murcia, Spain), which served as the central ethics committee for the study, approved the study (Internal Protocol Code: 2025-1-1 HCUVA).

Adult patients (age ≥ 18 years) with tumour infiltration of major vascular structures requiring vascular resection and subsequent reconstruction to achieve complete tumour removal and restore adequate hepatic inflow or outflow were included. Eligible procedures comprised hepatectomy with vascular reconstruction involving the inferior vena cava (IVC), right, middle, or left hepatic veins (HVs), the hepatocaval confluence, the portal vein (PV), or the hepatic artery (HA). The final surgical approach (*ante situm*, *ex situ*, or *in situ*) was determined according to intraoperative findings and the preference and expertise of the surgical team. A minimum postoperative follow-up of 6 months was also required. Patients with end-stage liver disease or pre-existing severe liver failure were excluded, as were those who underwent surgery with purely palliative intent.

Tumour data were collected across participating centres using multiple sources for the study period. For earlier patients, data were retrieved from institutional surgical registries, prospectively maintained hepatobiliary databases, operative reports, discharge summaries, and archived medical records that were subsequently reviewed and digitized. For more recent patients, comprehensive electronic medical records were used. Only variables that could be reliably and consistently assessed across centres and time periods were included in the analysis, and patients with insufficient documentation to accurately evaluate key variables or outcomes were excluded.

### Endpoints

The primary aim of this study was to evaluate postoperative outcomes in patients undergoing liver resection with vascular reconstruction for primary or secondary hepatobiliary tumours. Secondary aims included analysis of complication rates across different surgical approaches, the impact of vascular graft types and reconstruction sites, comparisons between perihilar cholangiocarcinoma (PHC) and other tumour entities, the identification of predictors of morbidity and mortality, and the evaluation of oncological outcomes at 1, 3, and 5 years.

### Study outcomes

The variables assessed included demographic data, co-morbidities, tumour characteristics, vascular involvement, preoperative treatments, surgical details, postoperative complications, and short- and long-term clinical outcomes. Demographic variables included age, sex, body weight and height, body mass index, American Society of Anesthesiologists grade, the Charlson co-morbidity index, and Eastern Cooperative Oncology Group performance status. Co-morbidities evaluated were diabetes, severe cardiac disease, chronic obstructive pulmonary disease, and chronic kidney disease (stage ≥ 3). Tumour-related variables included tumour type, location, the size of the largest lesion, and the number of tumours. Vascular infiltration was assessed by identifying the structures involved, including the IVC, HV, PV, and HA.

Preoperative treatments included chemotherapy (number of cycles), and locoregional therapies such as transarterial chemoembolization or radioembolization. Surgical variables included the approach (*in situ*, *ante situm*, *ex situ*), the use of hepatic perfusion and the type of preservation solution, the type of hepatectomy, the method of vascular occlusion (total, partial, or Pringle manoeuvre), the use of extracorporeal bypass, and the vascular reconstruction strategy, including graft type. Intraoperative blood loss, transfusion requirements, and operative time were recorded. Liver resections were classified according to the Brisbane 2000 Terminology of the International Hepato-Pancreato-Biliary Association (IHPBA)^11^.

Postoperative morbidity was assessed up to 90 days after surgery to capture clinically relevant complications beyond the index hospitalization. Complications were graded according to the Clavien–Dindo classification within 90 days and included bile leak, postoperative liver failure as defined by the International Study Group of Liver Surgery^12^, ascites, bleeding, and pulmonary, infectious, cardiovascular, cerebrovascular, and late biliary complications. The length of hospital stay (LOS) and 90-day readmission rates were recorded when reliably available. Follow-up data were obtained from institutional surgical databases, electronic medical records, and structured outpatient follow-up at participating tertiary referral centres, including patients referred from other institutions. Prophylactic and postoperative anticoagulation was also analysed. Oncological outcomes were evaluated through overall survival (OS) and disease-free survival (DFS), with time-to-recurrence analyses performed only in patients with malignant disease and stratified by primary tumour type; patients with non-malignant indications were excluded from oncological analyses.

### Statistical analysis

Continuous variables are presented as the median with interquartile range (i.q.r.), and categorical variables are summarized as absolute and relative frequencies. Continuous variables were compared between groups using the Kruskal–Wallis test. Categorical variables were compared using the χ^2^ test; however, Fisher’s exact test was used when expected frequencies were too low to meet the assumptions of the χ^2^ test.

Simple logistic regression analyses were conducted, and results are reported as log-odds coefficients along with their corresponding *P* values. For multivariable analysis, logistic regression models were constructed using a backward stepwise selection approach based on the Akaike information criterion. Variables that did not improve model fit were sequentially excluded, yielding a parsimonious final model. Statistical significance was defined as a *P* < 0.050.

The Fine–Gray model was used for survival analysis in the presence of competing risks, because mortality from unrelated causes could prevent the observation of the event of interest. Survival probabilities were estimated using the Kaplan–Meier method, and survival curves were generated to illustrate time-to-event distributions. Survival rates at 1, 3, and 5 years were calculated for both OS and DFS. Differences in survival were examined according to tumour type, and associations were further assessed using Cox proportional hazards regression.

## Results

### Overall cohort

Data for the 532 patients, classified according to the surgical technique used (*in situ*, 85.3% (454 patients); *ante situm*, 9.2% (49); *ex situ*, 5.5% (29)) are presented in *Table S1*. The median age of the overall cohort was 63 years and 50.4% (268) were female. The most common indications were colorectal liver metastases (CRLM; 30.3%, 161 patients), intrahepatic cholangiocarcinoma (iCCA; 26.5%, 141), PHC (19.9%, 106), and hepatocellular carcinoma (HCC; 8.6%, 46). The most frequently performed surgical procedures were extended right hepatectomy (24.8%, 132), right hepatectomy (23.9%, 127), and extended left hepatectomy (15.4%, 82). The median tumour size was 65 (i.q.r. 35–90) mm, with bilobar involvement in 38.7% (199) of patients. Liver hypertrophy techniques were required in 20.3% (108) of patients.

The median vascular occlusion time was 85 minutes (40-110 min), and 23.9% of patients required extracorporeal bypass. Intraoperative blood transfusion was needed in 50.7% of patients, with a median operative time of 390 (range 300–493) min. Major complications (Clavien–Dindo ≥ IIIa) occurred in 40.6% (n=216) of patients, and the overall 90-day mortality rate was 9.6% (52). The median LOS was 13 days, and the 90-day readmission rate was 17.2% (88).

### Infiltrated vascular structure

The most frequently tumour-involved vascular structure was the retrohepatic IVC, followed by the HV, PV, and HA. *Figure 1* shows the different combinations of tumour-involved vascular structures. In the multivariate analysis, IVC repair remained associated with a lower incidence of bile leakage, postoperative bleeding, pulmonary complications, and late biliary complications, although it was also linked to a greater need for intraoperative transfusion. HV reconstruction was independently associated with higher transfusion requirements, longer operative time, prolonged LOS and intensive care unit (ICU) stay, and a higher 90-day readmission rate. Conversely, PV reconstruction was significantly associated with higher rates of post-hepatectomy liver failure, ascites, postoperative infection, Clavien–Dindo ≥ IIIa complications, and prolonged operative time. HA reconstruction was associated with an increased risk of postoperative ascites, cardiovascular complications, and prolonged surgical time (*Table 1*).

**Fig. 1:**
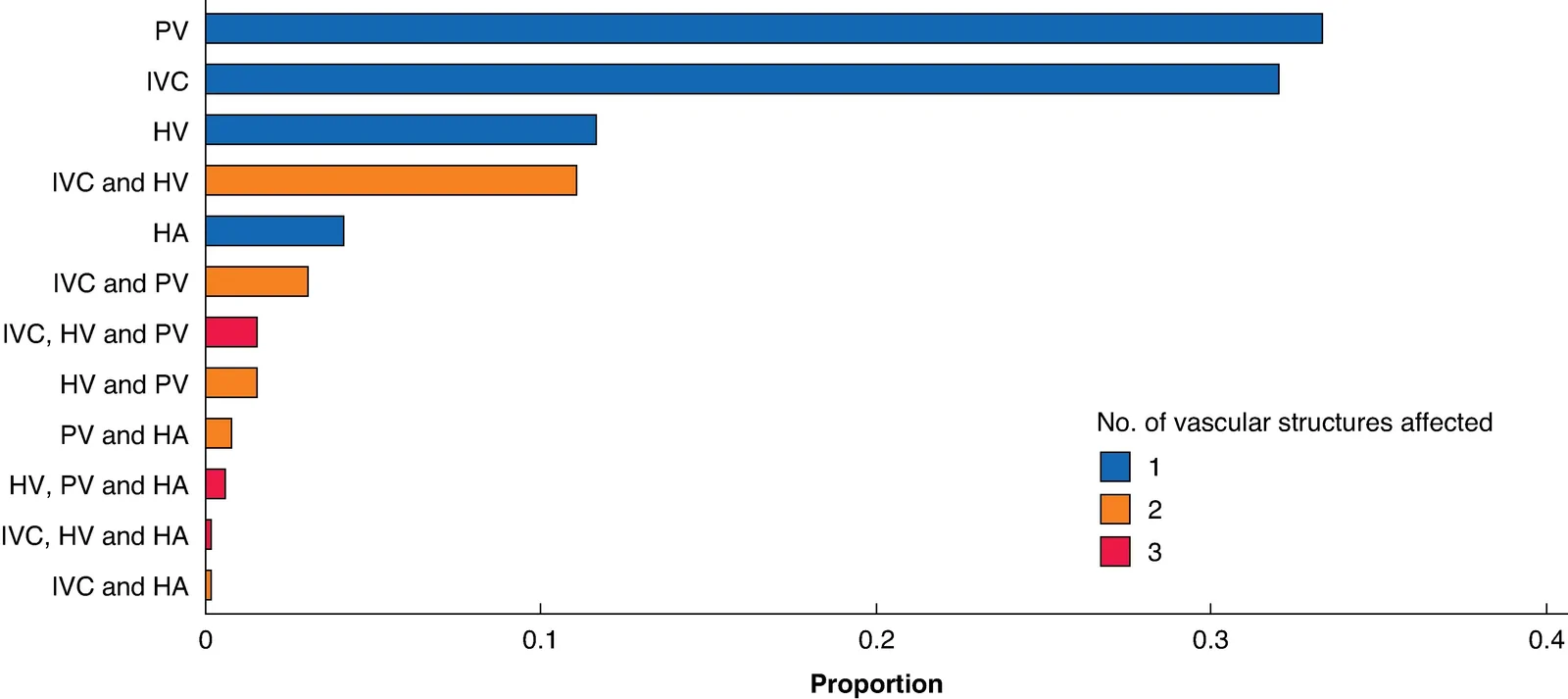
Distribution of exclusive combinations of vascular structures infiltrated by tumours PV, portal vein; IVC, inferior vena cava; HV, hepatic vein; HA, hepatic artery.

**Table 1:** Multiple logistic regression analysis of the association between vascular structure involvement and perioperative outcomes

	Proportion	IVC		Hepatic vein		Portal vein		Hepatic artery	
		Coefficient	*P*	Coefficient	*P*	Coefficient	*P*	Coefficient	*P*
**Multiple logistic regression (log odds)**									
Bile leakage	0.203	−0.887	< 0.001						
Liver failure	0.133					0.735	0.008		
Postoperative ascites	0.242					0.680	0.003	1.102	0.005
Postoperative bleeding	0.107	−1.416	< 0.001						
Pulmonary complications	0.194	−0.860	0.001						
Postoperative infection	0.254			0.364	0.145	0.897	< 0.001	0.762	0.061
Cardiovascular complications	0.055							1.386	0.021
Cerebrovascular complications	0.021								
Late biliary complications	0.053	−0.946	0.053						
Clavien–Dindo ≥ IIIa	0.406	−0.487	0.073			0.514	0.044		
Intraoperative transfusion	0.507	0.582	0.011	0.693	0.005				
Intraoperative blood loss > 718 ml	718 (500–1500) ml*	0.759	0.034	0.489	0.152	1.358	< 0.001		
Operation time > 390 min	390 (300–493) min*			0.848	0.002	0.847	0.001	0.991	0.046
ICU stay > 3 days	3 (1–7) days*			0.648	0.015			1.024	0.072
Length of hospital stay > 13 days	13 (9–21) days*			0.594	0.003				
Readmission within 90 days	0.172			0.592	0.037				

^*^Values are the median (interquartile range). IVC, inferior vena cava; min, minutes; ICU, intensive care unit.

### PHC *versus* primary/secondary tumours

When comparing the 106 patients with PHC to the 426 patients undergoing surgery for other indications (*Table 2*), significant differences were identified in many clinical and surgical variables. Patients with PHC were older (median age 67.5 *versus* 62 years; *P* = 0.001) and had a higher prevalence of severe heart disease (34% (36) *versus* 3.8% (16); *P* < 0.001), chronic obstructive pulmonary disease (10.4% (11) *versus* 1.9% (8); *P* < 0.001), and chronic kidney disease (13.2% (14) *versus* 1.9% (8); *P* < 0.001) than patients undergoing surgery for other indications. The PHC group underwent lymphadenectomy far more frequently (95.3% (101) *versus* 33.3% (140); *P* < 0.001) but required the use of extracorporeal bypass considerably less often (1.9% (2) *versus* 29.3% (125); *P* < 0.001). In addition, bile leak (41.5% (44 ) *versus* 15% (64); *P* < 0.001) and postoperative ascites (33% (35) *versus* 22.1% (94); *P* = 0.026) were more common in the PHC group. However, no significant differences were observed between the PHC and other surgical indications groups in terms of major complications (Clavien–Dindo ≥ IIIa; 38.7% (41) *versus* 41.1% (175), respectively; *P* = 0.734), mortality (8.5% (9) *versus* 9.9% (42), respectively; *P* = 0.807), LOS (13.5 *versus* 13 days, respectively; *P* = 0.518), or 90-day readmission rates (18.2% *versus* 17%, respectively; *P* = 0.894; *Table 2*).

**Table 2:** Baseline, surgical, and postoperative variables in patients with PHC or primary liver tumours or metastases

	PHC	Primary tumours and metastases	P*
No. of patients	106 (19.9%)	426 (80.1%)	
Age (years), median (i.q.r.)	67.5 (58–72)	62 (52–70)	< 0.001
**Sex**			0.186
Female	47 (44.3%)	222 (52.1%)	
Male	59 (55.7%)	204 (47.9%)	
**Type of hepatectomy**			
Extended right hepatectomy	36 (34%)	96 (22.5%)	< 0.001
Right hepatectomy	31 (29.2%)	96 (22.5%)	
Extended left hepatectomy	17 (16%)	65 (15.3%)	
Left hepatectomy	16 (15.1%)	44 (10.3%)	
Wedge resection	0	49 (11.5%)	
Other	6 (5.7%)	76 (17.8%)	
Malignant disease	106 (100%)	415 (97.4%)	0.132
Largest tumour size (mm), median (i.q.r.)	30 (20–40)	75 (45–100)	< 0.001
Hypertrophy technique	30 (28.3%)	78 (18.3%)	0.030
Liver perfusion	1 (0.9%)	121 (28.4%)	< 0.001
Vascular occlusion	83 (78.3%)	408 (95.8%)	< 0.001
Vascular occlusion time (min), median (i.q.r.)	65 (28–102)	85 (45–113)	0.037
Use of extracorporeal bypass	2 (1.9%)	125 (29.3%)	< 0.001
Lymphadenectomy	101 (95.3%)	140 (33.3%)	< 0.001
**Type of vascular repair**			
Primary suture	87 (82.1%)	149 (35%)	< 0.001
Synthetic graft	6 (5.7%)	173 (40.6%)	
Autologous graft	10 (9.4%)	53 (12.4%)	
Xenograft	1 (0.9%)	22 (5.2%)	
Allograft	1 (0.9%)	14 (3.3%)	
Other	1 (0.9%)	15 (3.5%)	
Intraoperative blood loss (ml), median (i.q.r.)	700 (500–1250)	750 (500–1800)	0.266
Intraoperative transfusion	34 (33%)	220 (55.3%)	< 0.001
Operation time (min), median (i.q.r.)	395 (329–493)	390 (299–493)	0.491
Prophylactic anticoagulation	72 (69.2%)	273 (67.6%)	0.838
Therapeutic anticoagulation	48 (49%)	166 (49.8%)	0.971
**Postoperative complications at 90 days**			
Bile leakage	44 (41.5%)	64 (15%)	< 0.001
Liver failure	18 (17%)	53 (12.4%)	0.285
Postoperative ascites	35 (33%)	94 (22.1%)	0.026
Postoperative bleeding	14 (13.2%)	43 (10.1%)	0.452
Pulmonary complications	22 (20.8%)	81 (19%)	0.780
Postoperative infection	31 (29.2%)	104 (24.4%)	0.362
Cardiovascular complications	3 (2.8%)	26 (6.1%)	0.246
Late biliary complications	9 (8.7%)	17 (4.4%)	0.145
Clavien–Dindo ≥ IIIa	41 (38.7%)	175 (41.1%)	0.734
Clavien–Dindo ≥ IIIb	31 (29.2%)	121 (28.4%)	0.959
Mortality	9 (8.5%)	42 (9.9%)	0.807
ICU stay (days), median (i.q.r.)	1 (0–5.5)	3 (2–8)	< 0.001
Length of hospital stay (days), median (i.q.r.)	13.5 (11–21)	13 (9–21)	0.518
Readmission within 90 days	18 (18.2%)	70 (17%)	0.894

Values are *n* (%) unless otherwise stated. PHC, perihilar cholangiocarcinoma; i.q.r., interquartile range; min, minutes; ICU, intensive care unit. *χ^2^ test or Fisher’s exact test and Mann–Whitney *U* test or Student’s *t* test.

*Table 3* includes data only for the 426 patients with primary liver tumours or metastases (excluding PHC). In this subgroup of patients, the *in situ* surgical approach was the most common (81.9%, 349), followed by *ante situm* (11.5%, 49) and *ex situ* (6.6%, 28). *Ante situm*/*ex situ* approaches were associated with younger age (*P* < 0.001), larger tumours (*P* < 0.001), higher rates of bilobar disease (*P* < 0.001), and extracorporeal bypass (*P* < 0.001). Consequently, these approaches had greater blood loss (*P* < 0.001), transfusion needs (*P* < 0.001), and longer operative times (*P* < 0.001). The *ante situm* and *ex situ* groups, compared with the *in situ* group, had significantly higher postoperative complications (Clavien–Dindo ≥ IIIa; 67.3% (33) and 64.3% (18) *versus* 35.5% (124), respectively; *P* < 0.001) and ICU stays (6.5 and 5 *versus* 3 days, respectively; *P* < 0.001). Mortality was also higher in the *ante situm* and *ex situ* groups than in the *in situ* group (16.3% (8) and 17.9% (5) *versus* 8.3% (29), respectively; *P* = 0.060). In this patient cohort (*Table S2*), multivariate analysis showed that the *in situ* approach was associated with a lower incidence of biliary and pulmonary complications, as well as a shorter operative time. Conversely, hypothermic perfusion of the remnant liver was associated with a higher rate of late biliary complications and major complications in general. Both partial and total vascular occlusion was associated with increased postoperative bleeding, Clavien–Dindo ≥ IIIa complications, and the need for intraoperative transfusion. Finally, the use of extracorporeal bypass was associated with increased blood loss requiring intraoperative transfusion, longer operative time, prolonged ICU stay, and higher hospital readmission rates. A detailed overview of these associations is shown in *Fig. 2*.

**Fig. 2:**
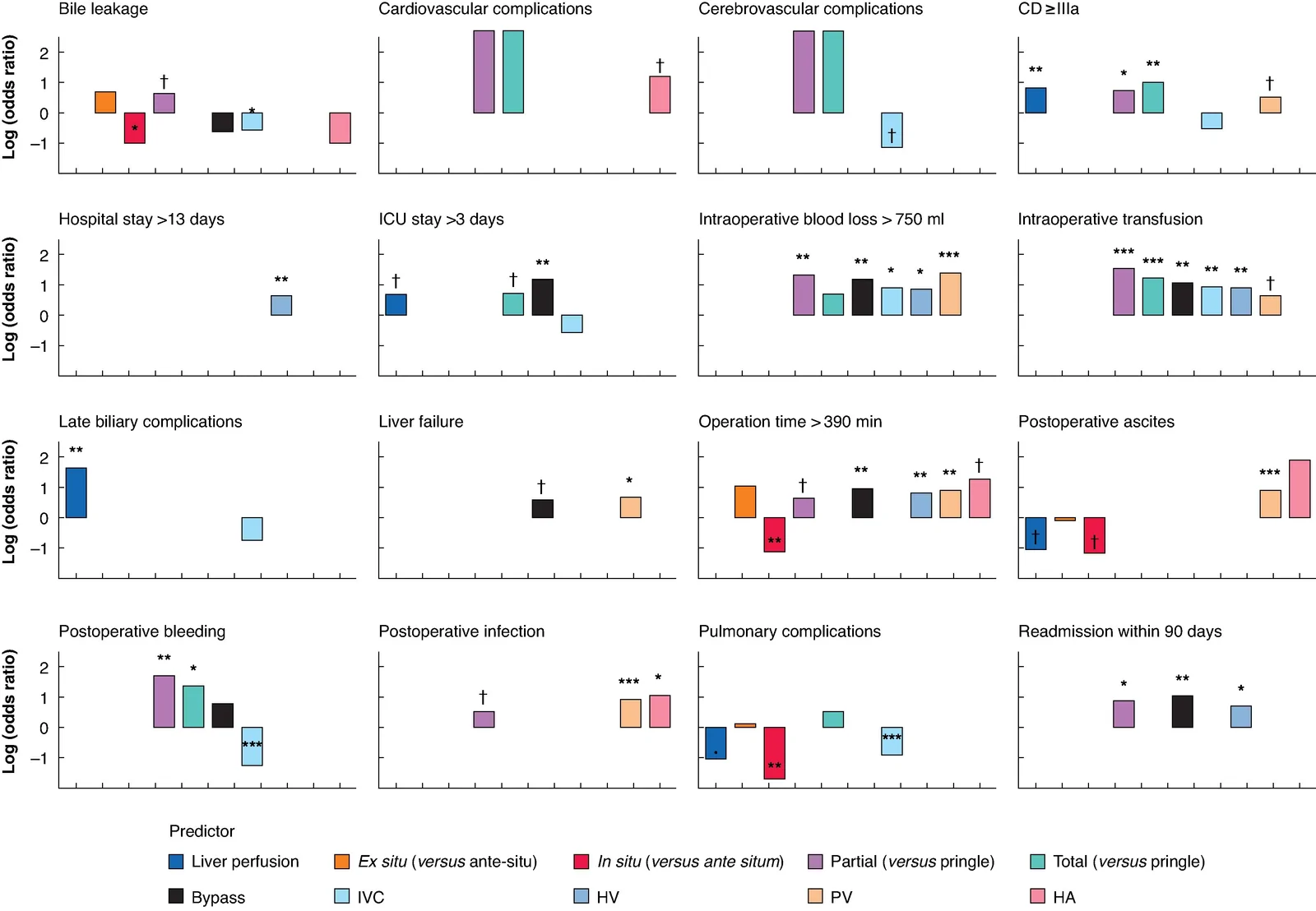
Separate multivariable logistic regression models for each postoperative complication, fitted using Akaike information criterion-based variable selection, identifying the most relevant predictors for each outcome Each panel represents one complication and shows the estimated log odds for the predictors retained in the final model. CD, Clavien–Dindo; ICU, intensive care unit; min, minutes; IVC, inferior vena cava; HV, hepatic vein; PV, portal vein; HA, hepatic artery. *P < 0.050, **P < 0.010, ***P < 0.001, †*P* < 0.100.

**Table 3:** Clinical and surgical variables in the entire study cohort and according to surgical approach in patients with primary tumours or metastases (excluding perihilar cholangiocarcinoma indication)

	Total (*n* = 426)	Surgical approach			P*
		*Ante situm* (*n* = 49)	*Ex situ* (*n* = 28)	*In situ* (*n* = 349)	
Age (years), median (i.q.r.)	62 (52–70)	57 (50–62)	55 (45.5–63)	63 (54–71)	< 0.001
**Type of hepatectomy**					
Extended right hepatectomy	96 (22.5%)	15 (30.6%)	11 (39.3%)	70 (20.1%)	< 0.001
Right hepatectomy	96 (22.5%)	6 (12.2%)	1 (3.6%)	89 (25.5%)	
Extended left hepatectomy	65 (15.3%)	9 (18.4%)	9 (32.1%)	47 (13.5%)	
Left hepatectomy	44 (10.3%)	2 (4.1%)	0	42 (12%)	
Wedge resection	49 (11.5%)	2 (4.1%)	0	47 (13.5%)	
Other	76 (17.8%)	15 (30.6%)	7 (25%)	54 (15.5%)	
**Type of primary tumour**					
CRLM	161 (37.8%)	8 (16.3%)	8 (28.6%)	145 (41.5%)	0.006
iCCA	141 (33.1%)	28 (57.1%)	9 (32.1%)	104 (29.8%)	
HCC	46 (10.8%)	5 (10.2%)	3 (10.7%)	38 (10.9%)	
Sarcoma	22 (5.2%)	3 (6.1%)	2 (7.1%)	17 (4.9%)	
Non-CRLM	23 (5.4%)	2 (4.1%)	4 (14.3%)	17 (4.9%)	
Other	33 (7.7%)	3 (6.1%)	2 (7.1%)	28 (8%)	
Malignant disease	415 (97.4%)	48 (98%)	27 (96.4%)	340 (97.4%)	0.828
Largest tumour size (mm), median (i.q.r.)	75 (45–100)	82 (60–100.5)	115 (70–150)	70 (41.5–90)	< 0.001
Hypertrophy technique	78 (18.3%)	8 (16.3%)	4 (14.3%)	66 (18.9%)	0.772
Liver perfusion	121 (28.4%)	48 (98%)	28 (100%)	45 (12.9%)	< 0.001
**Type of occlusion**					
Pringle	119 (29.2%)	3 (6.1%)	0	116 (35%)	< 0.001
Partial	94 (22.1%)	4 (8.2%)	3 (10.7%)	87 (24.9%)	
Total	195 (47.8%)	42 (85.7%)	25 (89.3%)	128 (38.7%)	
Vascular occlusion time (min), median (i.q.r.)	85 (45–113)	117.5 (89–146)	167 (115–225)	71 (36–100)	< 0.001
Use of extracorporeal bypass	125 (29.3%)	39 (79.6%)	22 (78.6%)	64 (18.3%)	< 0.001
Lymphadenectomy	140 (33.3%)	30 (65.2%)	9 (36%)	101 (28.9%)	< 0.001
**Type of vascular repair**					
Primary suture	149 (35%)	5 (10.2%)	2 (7.1%)	142 (40.7%)	< 0.001
Synthetic graft	173 (40.6%)	24 (49%)	21 (75%)	128 (36.7%)	
Autologous graft	53 (12.4%)	4 (8.2%)	1 (3.6%)	48 (13.8%)	
Xenograft	22 (5.2%)	1 (2%)	1 (3.6%)	20 (5.7%)	
Allograft	14 (3.3%)	6 (12.2%)	1 (3.6%)	7 (2%)	
Other	15 (3.5%)	9 (18.4%)	2 (7.1%)	4 (1.1%)	
Intraoperative blood loss (ml), median (i.q.r.)	750 (500–1800)	2500 (900–3700)	1500 (1000–3000)	600 (400–1375)	< 0.001
Intraoperative transfusion	220 (55.3%)	34 (79.1%)	20 (87%)	166 (50%)	< 0.001
Operation time (min), median (i.q.r.)	390 (299–493)	560 (425–634)	610 (540–765)	359 (270–447)	< 0.001
Prophylactic anticoagulation	273 (67.6%)	28 (71.8%)	18 (78.3%)	227 (66.4%)	0.419
Therapeutic anticoagulation	166 (49.8%)	28 (68.3%)	5 (31.2%)	133 (48.2%)	0.017
**Postoperative complications at 90 days**					
Bile leakage	64 (15%)	9 (18.4%)	8 (28.6%)	47 (13.5%)	0.083
Liver failure	53 (12.4%)	9 (18.4%)	4 (14.3%)	40 (11.5%)	0.311
Postoperative ascites	94 (22.1%)	10 (20.4%)	7 (25%)	77 (22.1%)	0.896
Postoperative bleeding	43 (10.1%)	9 (18.4%)	2 (7.1%)	32 (9.2%)	0.141
Pulmonary complications	81 (19%)	15 (30.6%)	9 (32.1%)	57 (16.3%)	0.011
Postoperative infection	104 (24.4%)	10 (20.4%)	9 (32.1%)	85 (24.4%)	0.513
Cardiovascular complications	26 (6.1%)	5 (10.2%)	2 (7.1%)	19 (5.4%)	0.307
Late biliary complications	17 (4.4%)	4 (8.5%)	3 (12%)	10 (3.2%)	0.036
Clavien–Dindo ≥ IIIa	175 (41.1%)	33 (67.3%)	18 (64.3%)	124 (35.5%)	< 0.001
Clavien–Dindo ≥ IIIb	121 (28.4%)	23 (46.9%)	17 (60.7%)	81 (23.2%)	< 0.001
Mortality	42 (9.9%)	8 (16.3%)	5 (17.9%)	29 (8.3%)	0.060
ICU stay (days), median (i.q.r.)	3 (2–8)	6.5 (3–10)	5 (3–10)	3 (1–5)	< 0.001
Length of hospital stay (days), median (i.q.r.)	13 (9–21)	15 (11–26)	15.5 (11–32.5)	13 (8–20)	0.015
Readmission within 90 days	70 (17%)	7 (14.6%)	8 (30.8%)	55 (16.3%)	0.171

Values are *n* (%) unless otherwise stated. i.q.r., interquartile range; CRLM, colorectal liver metastases; iCCA, intrahepatic cholangiocarcinoma; HCC, hepatocellular carcinoma; min, minutes; ICU, intensive care unit. *χ^2^ test or Fisher’s exact test and Mann–Whitney *U* test or Student’s *t* test.

### Vascular grafts

In all, 296 patients (55.6%) underwent vascular graft placement. The most frequently used grafts were synthetic (60.5%, 192), followed by autologous grafts (21.3%, 63) and xenografts (7.8%, 23). Synthetic grafts were more commonly used for IVC repair (*P* < 0.001), whereas autologous grafts and allografts were preferred in HV reconstructions (*P* < 0.001).

Graft-related complications were observed in 58 patients (22.3%; *Table S3*). Postoperative infectious complications were significantly associated with graft-related complications (40.3% (25) *versus* 23.8% (62) in the group without graft-related complications; *P* < 0.001), and bile leakage occurred more frequently among those with than without graft complications (42.1% (16) *versus* 14.6% (38), respectively; *P* = 0.003). Graft-related complications were more frequent in patients requiring PV (*P* = 0.019) or HA reconstruction (*P* = 0.010). Therapeutic postoperative anticoagulation (36% (40) *versus* 48.5% (111); *P* < 0.001), and prophylactic anticoagulation (10.2% (17) *versus* 68.7% (*n* = 167); *P* = 0.003) were less common in patients with than without graft-related complications.

### Temporal comparisons of surgical and postoperative outcomes

When comparing the two study periods (1990–2010 *versus* 2011–2024), several statistically significant differences were observed (*Table S4*). In the more recent period, patients presented with a significantly smaller median largest tumour size (60 *versus* 80 mm; *P* < 0.001) and a markedly higher use of liver hypertrophy techniques (23.6% (106) *versus* 2.4% (2); *P* < 0.001). Surgical strategy also evolved, with increased use of the Pringle manoeuvre (*P* < 0.001) and a shift in vascular reconstruction techniques towards primary suture repair (47.1% (212) *versus* 29.3% (24); *P* < 0.001), alongside reduced use of synthetic grafts. Intraoperative blood loss (700 *versus* 1000 ml; *P* = 0.007) and transfusion requirements (48.3% (211) *versus* 67.2% (43); *P* = 0.003) were significantly lower in the more recent period. Changes in anticoagulation practice were evident, with a reduction in the use of prophylactic anticoagulation (63% (273) *versus* 96% (72); *P* < 0.001) and an increase in the use of therapeutic anticoagulation (54.6% (208) *versus* 12% (6); *P* < 0.001) from 1990–2010 to 2011–2024. After surgery, the more recent period was associated with higher rates of Clavien–Dindo IIIa complications (*P* = 0.036), but no significant differences were observed in Clavien–Dindo IIIb complications, mortality, ICU stay, LOS, or 90-day readmission rates between the two periods.

### Oncological outcomes

Compared with the other indications, DFS according to the Kaplan–Meier method was significantly superior for HCC (*P* = 0.011), whereas according to the Fine–Gray model PHC was associated with a higher risk of recurrence in the presence of the competing risk of death (*P* = 0.022). Overall, respective DFS rates at 1, 3, and 5 years were 61%, 28%, and 24% for CRLM; 80.8%, 45.8%, and 45.8% for HCC; 69.5%, 34.7%, and 26.2% for iCCA; 51.3%, 22.4%, and 22.4% for non-CRLM; 52.1%, 19.1%, and 11% for PHC; and 63%, 44.1%, and 31.5% for sarcoma (*Fig. 3a*). Respective OS rates at 1, 3, and 5 years were 87.1%, 48.2%, and 29.7% for CRLM; 82.5%, 60.3%, and 42.7% for HCC; 84.3%, 53.3%, and 30.2% for iCCA; 75%, 46.7%, and 37.4% for non-CRLM; 76.2%, 36.3%, and 22.4% for PHC; and 87.4%, 87.4%, and 68.8% for sarcoma (*Fig. 3b*).

**Fig. 3:**
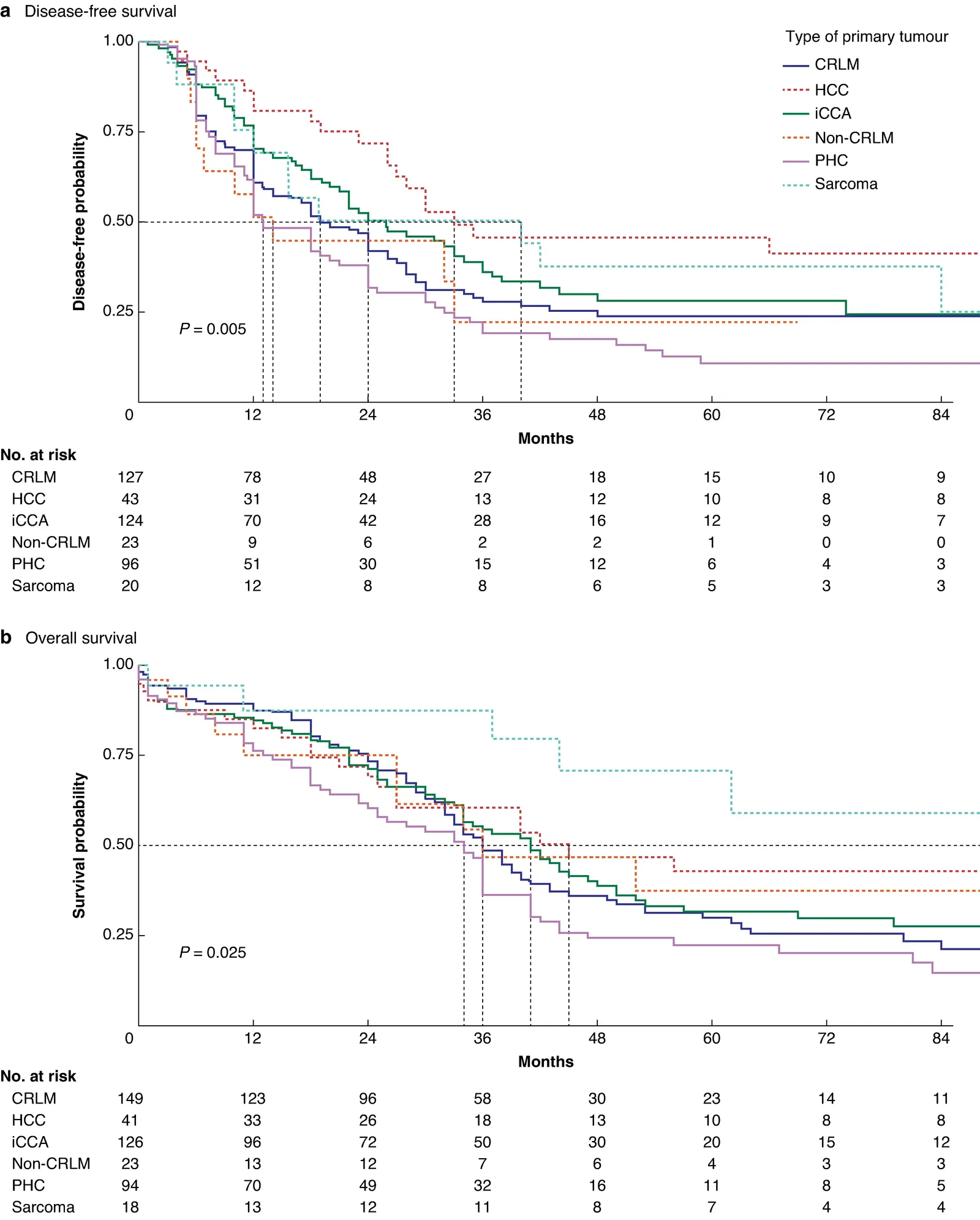
Kaplan–Meier survival curves stratified by type of primary tumour **a** Disease-free and **b** overall survival curves stratified by type of primary tumour. CRLM, colorectal liver metastases; HCC, hepatocellular carcinoma; iCCA, intrahepatic cholangiocarcinoma; PHC, perihilar cholangiocarcinoma.

## Discussion

This international multicentre study represents the largest and most comprehensive series to date evaluating the outcomes of extreme liver resections with vascular reconstruction in patients with advanced primary or metastatic hepatobiliary tumours involving major vascular structures. The findings confirm that these highly complex procedures are technically feasible and reproducible when performed by an experienced multidisciplinary team, although they remain associated with considerable morbidity and mortality.

There has been a longstanding debate over which surgical approach offers the best balance of safety and efficacy when hepatic resection with vascular reconstruction is required^3^. The present results support the preferential use of the *in situ* approach whenever technically feasible. In this cohort, *in situ* resections were associated with better perioperative outcomes, including lower rates of major complications, reduced blood loss and transfusion requirements, and shorter operative time and ICU stay compared with *ante situm* and *ex situ* procedures. The 90-day mortality observed for *ante situm* and *ex situ* procedures was higher than that reported in recent series from highly specialized centres^13,14^. These differences likely reflect the highly centralized expertise of such institutions, where these complex procedures are performed more frequently. In contrast, the present study represents a broader multicentre experience, suggesting that outcomes of extreme liver surgery procedures may be closely related to institutional experience and procedural volume.

Multiple vascular reconstruction techniques have been described in oncological liver surgery^2,15^. Synthetic grafts were predominantly used in IVC repairs, whereas autologous and allogeneic grafts were more frequently used for HV reconstruction. Synthetic grafts were considered not very suitable for HV and PV reconstructions, compared with IVC reconstructions, due to their rigidity. Despite these differences in anatomical application, no statistically significant association was observed between the type of graft material and the incidence of complications. This suggests that technical factors, patient-related variables, and the complexity of the vascular reconstruction itself likely play more critical roles in determining outcomes than the graft material used^16^. These data provide reassurance that graft selection and postoperative anticoagulation strategies can be adapted to individual patients and surgical characteristics without compromising safety.

Vascular involvement had a variable impact on surgical complexity and outcomes. IVC reconstruction, although most frequent, showed a more favourable perioperative profile and appeared technically more manageable with adequate vascular control. In contrast, HV reconstruction was more demanding, being associated with higher transfusion requirements, longer operative time, and prolonged hospital stay^17–19^. These findings suggest that the anatomical position of the HV, especially in proximity to the hepatocaval confluence, poses a greater technical challenge, increasing the likelihood of intraoperative bleeding and postoperative complications^20,21^.

PV reconstruction was associated with the worst intra- and postoperative outcomes, likely due to a higher risk of thrombosis and liver failure. However, this should not be interpreted as a direct causal effect, because PV involvement often reflects greater surgical complexity, including extensive hepatectomy, reduced future liver remnant, and possible biliary reconstruction. Therefore, PV reconstruction likely indicates a particularly high-risk surgical setting, highlighting the need for careful planning and thorough patient risk stratification^22,23^.

PHC poses significant management challenges, particularly when associated with vascular invasion^24,25^. These findings underscore that PHC requiring vascular reconstruction constitutes a clinically and surgically distinct entity compared with other liver tumours and metastases. Consequently, *in situ* approaches are favoured and complex perfusion strategies are generally unnecessary. Despite these anatomical and procedural differences, postoperative outcomes such as major complications, mortality, LOS, and readmission rates were comparable to those observed in other tumour types. However, the higher incidence of bile leaks and ascites in this group underscores the ongoing technical complexity of biliary with vascular reconstruction.

The role of liver graft perfusion during extreme liver surgery has traditionally been considered a protective measure to reduce ischaemic injury, particularly in prolonged resections^7,26^. However, the present study challenges this assumption. In subgroup analyses, hepatic perfusion was not associated with reduced late biliary complications or postoperative liver failure and was instead linked to higher rates of late biliary complications and major morbidity (Clavien–Dindo ≥ IIIa). This likely reflects its use in more complex, high-risk procedures rather than a true protective effect. Moreover, perfusion during *ante situm* resections is often suboptimal, frequently warmer than intended and without adequate oxygenation. With the development of flexible devices for hypothermic oxygenated perfusion, the impact of proper liver oxygenation warrants further evaluation^27,28^.

Similarly, the utility of extracorporeal bypass remains debatable^16,29–31^. Although extracorporeal bypass aims to maintain haemodynamic stability and reduce ischaemic injury during complex resections, this study showed its use was associated with higher transfusion requirements, longer operative time, prolonged ICU stay, and increased readmission rates. These findings question its routine use and support a more selective, individualized approach, reserving bypass and hepatic perfusion for patients where alternative vascular control is not feasible or safe.

A central question in the context of extreme liver surgery with vascular reconstruction is whether the morbidity and mortality rates associated with these high-risk procedures are justified by their oncological benefit^32,33^. In the present study, OS and DFS varied substantially across tumour types, reflecting both the heterogeneity of tumour biology and the limitations of resection-based strategies in certain settings. The most favourable long-term oncological outcomes were observed in HCC and sarcoma patients, with 5-year OS reaching 42.7% and 68.8%, respectively. These results suggest that, for select patients with good performance status and controlled disease burden, extreme liver resection can offer a meaningful chance of prolonged survival and may represent the best or only curative option when transplantation is not feasible.

In contrast, patients with CRLM and PHC showed poorer long-term DFS and OS, despite aggressive surgical management. For example, 5-year OS in CRLM patients was only 29.7%, and DFS dropped to 24%, raising legitimate concerns about the oncological durability of these procedures in this subgroup. Although PHC showed acceptable postoperative safety, its high recurrence rate and poor 5-year DFS raise concerns regarding the long-term benefit of resection, particularly when vascular reconstruction is required. However, recent evidence indicates that, despite the negative impact of vascular resection on OS compared with standard resections, it still provides a striking survival advantage over non-resection, suggesting that, in experienced hands, this approach may be justified for its oncological benefit^34^.

These findings reignite the discussion in the emerging field of transplant oncology^35^. In select tumours (particularly HCC, iCCA, PHC, and carefully selected CRLM), liver transplantation may provide better disease control and long-term survival while avoiding the burden of extreme surgery. These findings emphasize the importance of patient selection, tumour biology, and multidisciplinary decision-making. As transplant oncology evolves, studies comparing resection and transplantation will be essential to refine indications and optimize outcomes.

This study has limitations inherent to its retrospective design, including potential selection bias and heterogeneity in surgical techniques, perioperative management, and reporting standards across centres. The absence of centralized pathology review and standardized follow-up may have introduced variability in outcome assessment. Given the long inclusion period, advances in surgical techniques and perioperative care also may have influenced outcomes. A predefined time-based subanalysis was therefore performed to account for temporal changes in practice. Despite these limitations, this study represents the largest data set to date on extreme liver resections with vascular reconstruction, providing a comprehensive analysis of surgical complexity, postoperative outcomes, and long-term survival.

This international multicentre study shows that liver resection with vascular reconstruction is feasible but carries significant morbidity and mortality even in specialized centres. The choice between *in situ*, *ante situm*, and *ex situ* techniques may also reflect the experience and technical preference of each surgical team. These highly complex procedures are often tailored to the expertise of the operating centre and the familiarity of the surgical team with a given technique. Therefore, although the present study supports the preferential use of the *in situ* approach whenever technically feasible, the final choice of technique ultimately depends on the intraoperative findings and the preference and experience of the surgical team.

From an oncological perspective, outcomes largely depend on tumour biology. Although some tumour types derive limited benefit from resection, this may still exceed that of non-surgical management, underscoring the value of surgery despite its risks. These findings highlight the need for individualized patient selection and consideration of liver transplantation in select patients as transplant oncology advances. Prospective studies comparing resection and transplantation are required to define optimal strategies for advanced liver tumours with vascular involvement.

## Supplementary Material

zrag085_Supplementary_Data

## Data Availability

The data that support the findings of this study are available from the corresponding author upon reasonable request.

## References

[1] Azoulay D, Pascal G, Salloum C, Adam R, Castaing D, Tranecol N. Vascular reconstruction combined with liver resection for malignant tumours. Br J Surg 2013;100:1764–1775 10.1002/bjs.9295 24227362

[2] Radulova-Mauersberger O, Weitz J, Riediger C. Vascular surgery in liver resection. Langenbecks Arch Surg 2021;406:2217–2248 10.1007/s00423-021-02310-w PMC8578135 34519878

[3] Fard-Aghaie MH, Laengle J, Wagner KC, Reese T, Wirtz S, Oldhafer KJ. Liver surgery in the 2020s: *ante-situm* and *in-situ* resection are still indicated—a single-center study. HPB (Oxford) 2023;25:1030–1039 10.1016/j.hpb.2023.05.365 37328365

[4] Ichida A, Kokudo T, Shimada S, Hatano E, Kubo S, Kato Y et al Liver resection for hepatocellular carcinoma with tumor thrombus in the inferior vena cava or right atrium: a large-scale multicenter survey conducted in Japan. Ann Surg 2023;278:e549–e555 10.1097/SLA.0000000000005789 36591790

[5] Serrablo A, Giménez-Maurel T, Utrilla Fornals A, Serrablo L, Azoulay D. Current indications of ex-situ liver resection: a systematic review. Surgery 2022;172:933–942 10.1016/j.surg.2022.04.002 35791978

[6] Wong D, Hockley J, Parys S, Hodder R, Jansen S, Newman M. Current technology venous–venous bypass improves the safety of resection of sarcoma and benign retroperitoneal tumours involving the inferior vena cava. Eur J Vasc Endovasc Surg 2022;64:575–576 10.1016/j.ejvs.2022.07.049 35964890

[7] Li L, Hirukawa K, Morinaga J, Goto T, Isono K, Honda M et al Extreme surgery using the hypothermic perfusion technique for conventionally unresectable abdominal malignant tumours: a systematic review and meta-analysis. Eur J Surg Oncol 2025;51:109692 10.1016/j.ejso.2025.109692 40010010

[8] Aini A, Xiang C, Dong J. *In situ* liver resection under venovenous bypass (LR-VVB) or venoarterial bypass (LR-VAB) adjunct to total vascular exclusion (TVE) as extreme liver surgery. Hepatobiliary Surg Nutr 2025;14:506–510 10.21037/hbsn-2025-170 PMC12170105 40529927

[9] Azoulay D, Maggi U, Lim C, Malek A, Compagnon P, Salloum C et al Liver resection using total vascular exclusion of the liver preserving the caval flow, *in situ* hypothermic portal perfusion and temporary porta-caval shunt: a new technique for central tumors. Hepatobiliary Surg Nutr 2014;3:149–153 10.3978/j.issn.2304-3881.2014.05.02 PMC4073321 25019076

[10] Azoulay D, Lim C, Salloum C, Andreani P, Maggi U, Bartelmaos T et al Complex liver resection using standard total vascular exclusion, venovenous bypass, and *in situ* hypothermic portal perfusion: an audit of 77 consecutive cases. Ann Surg 2015;262:93–104 10.1097/SLA.0000000000000787 24950284

[11] Strasberg SM, Belghiti J, Clavien P-A, Gadzijev E, Garden JO, Lau WY et al The Brisbane 2000 terminology of liver anatomy and resections. HPB 2000;2:333–339 10.1080/136518202760378489 PMC2020531 18332933

[12] Rahbari NN, Garden OJ, Padbury R, Brooke-Smith M, Crawford M, Adam R et al Posthepatectomy liver failure: a definition and grading by the International Study Group of Liver Surgery (ISGLS). Surgery 2011;149:713–724 10.1016/j.surg.2010.10.001 21236455

[13] Weiner J, Hemming A, Levi D, Beduschi T, Matsumoto R, Mathur A et al *Ex vivo* liver resection and autotransplantation: should it be used more frequently? Ann Surg 2022;276:854–859 10.1097/SLA.0000000000005640 35920562

[14] Khan A, Fosby B, Yaqub S, Dueland S, Line PD. Surgical approaches for complex hepatic and perihepatic tumors: a single-center experience with *ante situm* and *ex situ* techniques. Int JSurg. 2026; DOI: 10.1097/JS9.0000000000004935 [Epub ahead of print] 10.1097/JS9.0000000000004935 41677321

[15] Lopez-Lopez V, Ferreras D, Eshmuminov D, Brusadin R, Robles-Campos R. The challenge of hepatic vein reconstruction in surgical oncology. Hepatobiliary Surg Nutr 2022;11:473–476 10.21037/hbsn-22-147 PMC9186207 35693399

[16] Mise Y, Hasegawa K, Satou S, Aoki T, Beck Y, Sugawara Y et al Venous reconstruction based on virtual liver resection to avoid congestion in the liver remnant. Br J Surg 2011;98:1742–1751 10.1002/bjs.7670 22034181

[17] Hemming AW, Reed AI, Langham MR, Fujita S, van der Werf WJ, Howard RJ. Hepatic vein reconstruction for resection of hepatic tumors. Ann Surg 2002;235:850–858 10.1097/00000658-200206000-00013 PMC1422515 12035042

[18] Wu CC, Peng CM, Cheng SB, Yeh DC, Lui WY, Liu TJ et al The necessity of hepatic vein reconstruction after resection of cranial part of the liver and major hepatic veins in cirrhotic patients. Surgery 2012;151:223–231 10.1016/j.surg.2010.10.014 21176933

[19] Terasaki F, Kaneoka Y, Maeda A, Takayama Y, Fukami Y, Takahashi T et al The impact of standardized methods of hepatic vein reconstruction with an external iliac vein graft. Hepatobiliary Surg Nutr 2021;10:163–171 10.21037/hbsn.2019.09.18 PMC8050583 33898557

[20] Zhou Y, Wu L, Xu D, Wan T, Si X. A pooled analysis of combined liver and inferior vena cava resection for hepatic malignancy. HPB (Oxford) 2017;19:768–774 10.1016/j.hpb.2017.05.008 28645571

[21] Oldhafer F, Ringe KI, Timrott K, Kleine M, Beetz O, Ramackers W et al Modified *ante situm* liver resection without use of cold perfusion nor veno-venous bypass for treatment of hepatic lesions infiltrating the hepatocaval confluence. Langenbecks Arch Surg 2018;403:379–386 10.1007/s00423-018-1658-1 29470630

[22] Liang L, Chen TH, Li C, Xing H, Han J, Wang MD et al A systematic review comparing outcomes of surgical resection and non-surgical treatments for patients with hepatocellular carcinoma and portal vein tumor thrombus. HPB (Oxford) 2018;20:1119–1129 10.1016/j.hpb.2018.06.1804 30056066

[23] Kim KB, Choi DW, Heo JS, Han IW, Shin SH, You Y et al The impact of portal vein resection on outcome of hilar cholangiocarcinoma. Ann Hepatobiliary Pancreat Surg 2021;25:221–229 10.14701/ahbps.2021.25.2.221 PMC8180392 34053925

[24] Pfister M, Ratti F, Gores GJ, Lesurtel M, Chiche L, Ebata T et al Recommendations on perihilar cholangiocarcinoma. The Milan Jury-based consensus. Ann Surg 2025; DOI: 10.1097/SLA.0000000000006773 [Epub ahead of print] 10.1097/SLA.0000000000006773 40478744

[25] Higuchi R, Yazawa T, Uemura S, Izumo W, Ota T, Kiyohara K et al Surgical outcomes for perihilar cholangiocarcinoma with vascular invasion. J Gastrointest Surg 2019;23:1443–1453 10.1007/s11605-018-3948-x 30203230

[26] Dubay D, Gallinger S, Hawryluck L, Swallow C, McCluskey S, McGilvray I. *In situ* hypothermic liver preservation during radical liver resection with major vascular reconstruction. Br J Surg 2009;96:1429–1436 10.1002/bjs.6740 19918862

[27] Cillo U, Furlanetto A, Nieddu E, Polacco M, Boetto R, Bassi D et al *Ante situm* liver surgery using machine perfusion liver preservation: pilot human experience. Br J Surg 2021;108:e235–e236 10.1093/bjs/znab095 33824957

[28] Schlegel A, Sakuraoka Y, Motwani K, Gourevitch D, Sharif K, Isaac J et al Outcome after *ex situ* or *ante situm* liver resection with hypothermic perfusion and auto-transplantation: a single-centre experience in adult and paediatric patients. J Surg Oncol 2020;122:1122–1131 10.1002/jso.26116 32705679

[29] Zhang KM, Hu XW, Dong JH, Hong ZX, Wang ZH, Li GH et al *Ex-situ* liver surgery without veno-venous bypass. World J Gastroenterol 2012;18:7290–7295 10.3748/wjg.v18.i48.7290 PMC3544032 23326135

[30] Cheng F, Yang Z, Zeng J, Gu J, Cui J, Ning J et al Anesthesia management of modified *ex vivo* liver resection and autotransplantation. Ann Transplant 2018;23:274–284 10.12659/AOT.907796 PMC6248320 29700275

[31] Lopez-Lopez V, Garcia-Lopez J, Eshmuminov D, Brusadin R, Lopez-Conesa A, Martinez-Insfran L et al Left renal vein graft and in situ hepatic perfusion in hepatectomy for complete tumor invasion of hepatic veins: hemodynamic optimization and surgical technique. Langenbecks Arch Surg 2022;407:1–7 10.1007/s00423-022-02451-6 PMC9283147 35102435

[32] Codony C, López-Ben S, Albiol M, Falgueras L, Castro E, Codina-Barreras A et al Extreme liver surgery as treatment of liver tumors involving the hepatocaval confluence. Clin Transl Oncol 2016;18:1131–1139 10.1007/s12094-016-1495-z 26960560

[33] Araujo RL, Linhares MM. Pushing the limits of liver surgery for colorectal liver metastases: current state and future directions. World J Gastrointest Surg 2019;11:34–40 10.4240/wjgs.v11.i2.34 PMC6397797 30842810

[34] Dong Y, Li Z, Podrascanin V, Eaton JE, Ilyas SI, Gores GJ et al Liver resection with and without vascular resection *versus* transplantation for *de novo* perihilar cholangiocarcinoma. Hepatology 2025;83:1128–1142 10.1097/HEP.0000000000001449 PMC13089817 40622853

[35] Sapisochin G, Hibi T, Toso C, Man K, Berenguer M, Heimbach J et al Transplant oncology in primary and metastatic liver tumors: principles, evidence, and opportunities. Ann Surg 2021;273:483–493 10.1097/SLA.0000000000004071 33065633

