# Supplementary material for: Predictors of morbidity and oncological benefit after liver resection with vascular reconstruction: international collaborative study from expert centres
Source: BJS Open. 2026 Sep 28;10(5):zrag085. doi: 10.1093/bjsopen/zrag085 (PMC13617666; doi:10.1093/bjsopen/zrag085)
Supplement: Supplementary file 1 [file zrag085_supplementary_data.docx]

**Predictors of Morbidity and Oncologic Benefit after Liver Resection with Vascular Reconstruction: An International Collaborative Study from Expert Centres**

Victor Lopez-Lopez^1^, Francesca Ratti^2,3^, Cecilia Maina^2,3^, Juan Gonzalez-Ferez^4^, Sarah Daniels^5^, Nikhil Suresh^5^, Safi Dokmak^6,7,8^, Elias Karam^6,7^, Augustin Suffisseu^6^, Yawen Dong^9^, Zhihao Li^10^, Rory L. Smoot^9^, Muhammad Ahmad Nadeem^11,12^, Federico Aucejo^11,12^, Emilio Vicente^13^, Enrico Gringeri^14^, Eleonora Nieddu^14^, Jordi Navinés^15^, Lucia Hernandez^1^, Mohammad H Fard-Aghaie^16^, Jens Rolinger^17^ , Leyre Serrablo^18^, Sanjay Govil^19^, William Archie^20^, Dionisios Vrochides^20^, Martin de Santibañes^21^, Eduardo de Santibañes^21^, Fabrizio Di Benedetto^22^, Silvio M P Balzan^23^, Cristina Dopazo^24^, Vicente Borrego Estella^25^, Giuseppe Kito Fusai^26^, Fernando Rotellar Sastre^27^, Roberto I Troisi^28^, Alejandro Serrablo^18^, Silvio Nadalin^17^, Karl J Oldhafer^16^, Esteban Cugat^15^, Umberto Cillo^14^, Andrea Schlegel^11,12^, Patrick Starlinger^9^, Mickael Lesurtel^6,7^, Luca Aldrighetti^2,3^, Pierre Alain Clavien^29^, Daniel Azoulay^30^, Peter Lodge^5*^, Ricardo Robles Campos^1*^

1. Department of Surgery and Transplantation, Virgen de la Arrixaca Clinic and University Hospital, IMIB-Pascual Parrilla, Murcia, Spain.
2. Hepatobiliary Surgery Division, IRCCS San Raffaele Scientific Institute, Milano, Italy.
3. Vita Salute San Raffaele University, Milano, Italy.
4. Group of Applied Mathematics in Science and Engineering, Faculty of Computer Science, University of Murcia, Spain.
5. HPB and Transplant Unit, St James's University Hospital, Leeds Teaching Hospitals NHS Trust, Leeds, United Kingdom.
6. Department of HPB Surgery and Liver Transplantation, Beaujon Hospital, Assistance Publique - Hôpitaux de Paris, Clichy, France.
7. University Paris Cité, Paris.
8. Centre de Recherche sur l’nflammation, INSERM Unité 1149, Clichy, France.
9. Department of Surgery, Division of Hepatobiliary and Pancreas Surgery, Mayo Clinic, Rochester, MN, USA.
10. William J. von Liebig Center for Transplantation and Clinical Regeneration, Mayo Clinic, Rochester, Minnesota, USA.
11. Department of Inflammation & Immunity, Lerner Research Institute, Cleveland Clinic, Cleveland, OH, USA
12. Transplantation Center, Digestive Disease and Surgery Institute, Cleveland Clinic, Cleveland, OH, USA.
13. General Surgery Department, Sanchinarro University Hospital, Instituto de Investigación Sanitaria HM Hospitales, Jose Camilo Cela University, Madrid, Spain.
14. Hepato-bilio-pancreatic surgery and Liver Transplantation Unit, Padova University Hospital, Padova, Italy
15. Department of General and Digestive Surgery, Hospital Germans Trias i Pujol, Badalona, Barcelona, Spain.
16. Semmelweis University Budapest, Asklepios Campus Hamburg. Department of Surgery, Division of HPB-Surgery, Asklepios Hospital Barmbek, Hamburg, Germany.
17. Department of General, Visceral, and Transplant Surgery, University Hospital Tuebingen, Tuebingen, Germany.
18. HPB Surgical Division, Miguel Servet University Hospital, Zaragoza, Spain.
19. Clinical Lead and Sr Consultant Hepatobiliary Surgery and Liver Transplantation, Apollo Hospitals, Bangalore, India.
20. Division of Abdominal Transplantation, Carolinas Medical Center, Atrium Health,Wake Forest University School of Medicine,Charlotte, NC, USA.
21. Department of General Surgery, Hepato-Pancreato-Biliary and Liver Transplant Unit, Hospital Italiano de Buenos Aires - Buenos Aires (Argentina).
22. Hepato-Pancreato-Biliary Surgery and Liver Transplantation Unit, University Hospital of Modena "policlinico", University Of Modena And Reggio Emilia - Modena (Italy).
23. Department of Surgery, Hospital Moinhos de Vento, Porto Alegre (Brazil) and Hepatobiliary and Pancreatic Surgery Center, Saint Gallen Clinic, Santa Cruz do Sul (Brazil).
24. Departamento de Cirugía HBP y Trasplante Hepático, Hospital Universitari Vall D´hebron, Universidad Autónoma de Barcelona - Barcelona (Spain).
25. General and Digestive Surgery Department, HCU Lozano Blesa of Zaragoza, Hospital Clínico Universitario Lozano Blesa, Calle San Juan Bosco, 15, 50009, Saragossa, Spain.
26. HPB & Liver Transplant Unit. Royal Free Hospital London – UK.
27. Department of General Surgery, Clínica Universidad de Navarra, University of Navarra,Pamplona, Spain.
28. Department of Clinical Medicine and Surgery, Division of HPB, Minimally Invasive and Robotic Surgery, Transplantation Center, Federico II University Hospital, 80131 Naples, Italy.
29. Department of Surgery and Transplantation, University of Zurich, Zurich, Switzerland.
30. Centre Hépato-Biliaire, Department of Surgery, Hôpital Paul Brousse, Assistance Publique-Hôpitaux de Paris, Villejuif, France.

*RRC and PL shared senior authorship.

 Corresponding Author:

Víctor López-López, MD, PhD

Department of General, Visceral and Transplantation Surgery

Clinic and University Hospital Virgen de la Arrixaca, Murcia

Phone: +34 968 36 95 00

E-mail: victorrelopez@gmail.es

**Supplementary Materials - Index**

| **Supplementary Tables** |  |
| --- | --- |
| Supplementary table 1 | *page 8* |
| Supplementary table 2 | *page 10* |
| Supplementary table 3 | *page 10* |
| Supplementary table 3 | *page 11* |

**Supplementary Tables**

**Supplementary Table 1.** Baseline characteristics, intraoperative variables, and postoperative outcomes according to surgical approach (in-situ, ante-situ, and ex-situ).

| **Variable** | **Overall (n=532)** | **Ante-situ (n=48)** | **Ex-situ (n=29)** | **In-situ (n=455)** | **p** |
| --- | --- | --- | --- | --- | --- |
| **Age, median (IQR)** | 532 | 49 (9.2%) | 29 (5.5%) | 454 (85.3%) |  |
| **Gender, female** | 63 (54-71) | 57 (50-62) | 55 (46-63) | 65 (55-72) | <0.001 |
| **ASA score** | 269 (50.6%) | 30 (61.2%) | 17 (58.6%) | 222 (48.9%) | 0.175 |
| <3 |  |  |  |  |  |
| ≥3 | 35 (7.1%) | 5 (10.2%) | 5 (17.2%) | 25 (6%) | 0.041 |
| **BMI, median (IQR)** | 460 (92.9%) | 44 (89.8%) | 24 (82.8%) | 392 (94%) |  |
| **Charlson Comorbidity Index, median (IQR)** | 24.9 (22.7-28.1) | 26 (23.6-28.5) | 27.6 (22.6-31.2) | 24.8 (22.6-27.9) | 0.113 |
| **ECOG performance status** | 5 (3-7) | 4.5 (3-6) | 5 (3-6.5) | 6 (3-8) | 0.19 |
| 0 |  |  |  |  |  |
| 1 | 214 (54.2%) | 33 (67.3%) | 19 (65.5%) | 162 (51.1%) | 0.016 |
| 2 | 139 (35.2%) | 8 (16.3%) | 8 (27.6%) | 123 (38.8%) | 0.016 |
| **Diabetes** | 42 (10.6%) | 8 (16.3%) | 2 (6.9%) | 32 (10.1%) | 0.016 |
| **Severe cardiac disease** | 92 (17.4%) | 6 (12.2%) | 2 (6.9%) | 84 (18.7%) | 0.163 |
| **COPD** | 52 (9.8%) | 2 (4.1%) | 1 (3.4%) | 49 (10.9%) | 0.215 |
| **Chronic renal failure** | 19 (3.6%) | 1 (2%) | 0 (0%) | 18 (4%) | 0.772 |
| **Type of hepatectomy** | 22 (4.2%) | 1 (2%) | 1 (3.4%) | 20 (4.4%) | 0.891 |
| Extended right hepatectomy |  |  |  |  |  |
| Right hepatectomy | 132 (24.8%) | 15 (30.6%) | 11 (37.9%) | 106 (23.3%) | <0.001 |
| Extended left hepatectomy | 127 (23.9%) | 6 (12.2%) | 1 (3.4%) | 120 (26.4%) | <0.001 |
| Left hepatectomy | 82 (15.4%) | 9 (18.4%) | 9 (31%) | 64 (14.1%) | <0.001 |
| Wedge resection | 60 (11.3%) | 2 (4.1%) | 0 (0%) | 58 (12.8%) | <0.001 |
| Other | 49 (9.2%) | 2 (4.1%) | 0 (0%) | 47 (10.4%) | <0.001 |
| **Type of primary tumor** | 82 (15.4%) | 15 (30.6%) | 8 (27.6%) | 59 (13%) | <0.001 |
| CRLM |  |  |  |  |  |
| iCCA | 161 (30.3%) | 8 (16.3%) | 8 (27.6%) | 145 (31.9%) | <0.001 |
| PHC | 141 (26.5%) | 28 (57.1%) | 9 (31%) | 104 (22.9%) | <0.001 |
| HCC | 106 (19.9%) | 0 (0%) | 1 (3.4%) | 105 (23.1%) | <0.001 |
| Sarcoma | 46 (8.6%) | 5 (10.2%) | 3 (10.3%) | 38 (8.4%) | <0.001 |
| Non-CRLM | 22 (4.1%) | 3 (6.1%) | 2 (6.9%) | 17 (3.7%) | <0.001 |
| Other | 23 (4.3%) | 2 (4.1%) | 4 (13.8%) | 17 (3.7%) | <0.001 |
| **Malignant** | 33 (6.2%) | 3 (6.1%) | 2 (6.9%) | 28 (6.2%) | <0.001 |
| **Largest tumor size (mm), median (IQR)** | 521 (97.9%) | 48 (98%) | 28 (96.6%) | 445 (98%) | 0.62 |
| **Bilobar disease** | 65 (35-90) | 82 (60-100.5) | 110 (70-150) | 57 (32-88) | <0.001 |
| **Previous hepatectomy** |  |  |  |  |  |
| **Preoperative chemotherapy** | 199 (38.7%) | 35 (71.4%) | 27 (93.1%) | 137 (31.4%) | <0.001 |
| **Need for regeneration technique** | 30 (5.6%) | 3 (6.1%) | 3 (10.3%) | 24 (5.3%) | 0.383 |
| **Liver perfusion** | 228 (43%) | 27 (56.2%) | 14 (50%) | 187 (41.2%) | 0.1 |
| **Vascular occlusion** | 108 (20.3%) | 8 (16.3%) | 4 (13.8%) | 96 (21.1%) | 0.492 |
| **Type of occlusion** | 122 (22.9%) | 48 (98%) | 29 (1.000) | 45 (9.9%) | <0.001 |
| Pringle | 491 (92.3%) | 49 (1.000) | 29 (1.000) | 413 (91%) | 0.013 |
| Partial |  |  |  |  |  |
| Total | 169 (34.4%) | 3 (6.1%) | 0 (0%) | 166 (40.2%) | <0.001 |
| **Time of vascular occlusion (min), median (IQR)** | 122 (24.8%) | 4 (8.2%) | 3 (10.3%) | 115 (27.8%) | <0.001 |
| **Extracorporeal bypass** | 200 (40.7%) | 42 (85.7%) | 26 (89.7%) | 132 (32%) | <0.001 |
| **Lymphadenectomy** | 85 (40-110) | 117.5 (89-146) | 163.5 (115-221) | 68 (35-100) | <0.001 |
| **Type of vascular repair** | 127 (23.9%) | 39 (79.6%) | 23 (79.3%) | 65 (14.3%) | <0.001 |
| Primary suture | 241 (45.8%) | 30 (65.2%) | 10 (38.5%) | 201 (44.3%) | 0.019 |
| Synthetic graft |  |  |  |  |  |
| Autologous graft | 236 (44.4%) | 5 (10.2%) | 2 (6.9%) | 229 (50.4%) | <0.001 |
| Xenograft | 179 (33.6%) | 24 (49%) | 21 (72.4%) | 134 (29.5%) | <0.001 |
| Allograft | 63 (11.8%) | 4 (8.2%) | 1 (3.4%) | 58 (12.8%) | <0.001 |
| Combined grafts | 23 (4.3%) | 1 (2%) | 1 (3.4%) | 21 (4.6%) | <0.001 |
| **Intraoperative blood loss (ml), median (IQR)** | 15 (2.8%) | 6 (12.2%) | 1 (3.4%) | 8 (1.8%) | <0.001 |
| **Intraoperative transfusion** | 16 (3%) | 9 (18.4%) | 3 (10.3%) | 4 (0.9%) | <0.001 |
| **Operation time (min), median (IQR)** | 718 (500-1500) | 2500 (900-3700) | 1450 (925-2925) | 650 (400-1350) | <0.001 |
| **Prophylactic postoperative anticoagulation** | 254 (50.7%) | 34 (79.1%) | 21 (87.5%) | 199 (45.9%) | <0.001 |
| **Therapeutic postoperative anticoagulation** | 390 (300-493) | 560 (425-634) | 638 (541-757) | 360 (283-454) | <0.001 |
| **Postoperative outcomes** | 345 (67.9%) | 28 (71.8%) | 19 (79.2%) | 298 (67%) | 0.397 |
| Bile leakage | 214 (49.7%) | 28 (68.3%) | 6 (35.3%) | 180 (48.3%) | 0.025 |
| Liver failure | 108 (20.3%) | 9 (18.4%) | 9 (31%) | 90 (19.8%) | 0.326 |
| Postoperative ascites | 71 (13.3%) | 9 (18.4%) | 4 (13.8%) | 58 (12.8%) | 0.473 |
| Postoperative bleeding | 129 (24.2%) | 10 (20.4%) | 8 (27.6%) | 111 (24.4%) | 0.749 |
| Pulmonary complications | 57 (10.7%) | 9 (18.4%) | 3 (10.3%) | 45 (9.9%) | 0.19 |
| Postoperative infection | 103 (19.4%) | 15 (30.6%) | 10 (34.5%) | 78 (17.2%) | 0.008 |
| Cardiovascular complications | 135 (25.4%) | 10 (20.4%) | 10 (34.5%) | 115 (25.3%) | 0.373 |
| Cerebrovascular complications | 29 (5.5%) | 5 (10.2%) | 2 (6.9%) | 22 (4.8%) | 0.222 |
| Late biliary complications | 11 (2.1%) | 2 (4.1%) | 1 (3.4%) | 8 (1.8%) | 0.248 |
| **Clavien ≥ IIIa** | 26 (5.3%) | 4 (8.5%) | 3 (11.5%) | 19 (4.6%) | 0.113 |
| **Clavien ≥ IIIb** | 216 (40.6%) | 33 (67.3%) | 19 (65.5%) | 164 (36.1%) | <0.001 |
| **Mortality** | 152 (28.6%) | 23 (46.9%) | 18 (62.1%) | 111 (24.4%) | <0.001 |
| **ICU stay (days), median (IQR)** | 51 (9.6%) | 8 (16.3%) | 5 (17.2%) | 38 (8.4%) | 0.055 |
| **Hospital stay (days), median (IQR)** | 3 (1-7) | 6.5 (3-10) | 5 (3-10) | 2 (0-5) | <0.001 |
| **Readmission within 90 days** | 13 (9-21) | 15 (11-26) | 16 (12-34) | 13 (9-21) | 0.016 |
| **Adjuvant chemotherapy** | 88 (17.2%) | 7 (14.6%) | 9 (33.3%) | 72 (16.5%) | 0.093 |

BMI: body mass index; CCR: colorectal cancer; iCCA: intrahepatic cholangiocarcinoma; PHC: perihiliar cholangiocarcinoma; HCC: hepatocelular carcinoma; COPD: Chronic obstructive pulmonary disease; F: female, IQR: interquarile range.* Percentages are calculated based on available data for each variable; denominators may differ due to missing values.

| **Simple Logistic regression (Log odds)** |  |  | **Approach type** | | | | |  |  | **Type of occlusion** | | | | |  |  |
| --- | --- | --- | --- | --- | --- | --- | --- | --- | --- | --- | --- | --- | --- | --- | --- | --- |
|  | 426 |  | **ante-situ** | **ante-situ** | | **in-situ** | | **Liver Perfussion** | | **Pringle** | **Partial** | | **Total** | | **Extracorporal bypass** | |
|  | n | prop |  | coef. | p | coef. | p | coef | p | ref | coef | p | coef | p | coef | p |
| Bile Leakage | 64 | 0,154 | ref. | 0.550 | 0.325 | -0.364 | 0.365 | 0.122 | 0.679 | ref. | 0.836 | 0.032 | 0.194 | 0.592 | -0.382 | 0.228 |
| Liver failure | 53 | 0,127 | ref. | -0.325 | 0.619 | -0.549 | 0.177 | 0.061 | 0.850 | ref. | 0.401 | 0.367 | 0.424 | 0.263 | 0.412 | 0.179 |
| Postoperative ascites | 94 | 0,226 | ref. | 0.236 | 0.674 | 0.107 | 0.778 | -0.525 | 0.060 | ref. | -0.028 | 0.930 | -0.746 | 0.010 | -0.647 | 0.022 |
| Postoperative bleeding | 43 | 0,103 | ref. | -1.099 | 0.181 | -0.798 | 0.0538 | 0.525 | 0.114 | ref. | 1.477 | 0.013 | 1.267 | 0.023 | 0.486 | 0.144 |
| Pulmonary complications | 81 | 0,195 | ref. | 0.041 | 0.936 | -0.814 | 0.018 | 0.248 | 0.349 | ref. | 0.116 | 0.756 | 0.272 | 0.377 | 0.134 | 0.616 |
| Postoperative Infection | 104 | 0,25 | ref. | 0.588 | 0.275 | 0.236 | 0.530 | -0.273 | 0.29 | ref. | 0.279 | 0.371 | -0.371 | 0.184 | -0.453 | 0.085 |
| Cardiovascular complications | 27 | 0,064 | ref. | -0.413 | 0.636 | -0.633 | 0.228 | 0.734 | 0.069 | ref. | 16.93 | 0.987 | 17.27 | 0.986 | 0.858 | 0.032 |
| Cerebrovascular complications | 9 | 0,022 | ref. | -0.160 | 0.898 | -0.884 | 0.288 | 1.143 | 0.093 | ref. | 17.22 | 0.992 | 17.12 | 0.992 | 1.107 | 0.103 |
| Late biliary complications | 17 | 0,045 | ref. | 0.406 | 0.615 | -0.983 | 0.109 | 0.978 | 0.050 | ref. | 0.599 | 0.554 | 1.049 | 0.185 | 0.769 | 0.123 |
| Clavien ≥3a | 176 | 0,413 | ref. | -0.201 | 0.690 | -1.376 | <0.001 | 1.116 | <0.001 | ref. | 0.514 | 0.090 | 1.139 | <0.001 | 0.851 | <0.001 |
| Intraoperative transfusion | 218 | 0,549 | ref. | 0.629 | 0.386 | -1.275 | <0.001 | 1.537 | <0.001 | ref. | 1.850 | <0.001 | 2.261 | <0.001 | 1.778 | <0.001 |
| Intraoperative blood loss (ml) >718 | 328 | 750 (500-1800) | ref. | 0.523 | 0.473 | -1.587 | <0.001 | 1.532 | <0.001 | ref. | 1.366 | <0.001 | 1.776 | <0.001 | 1.815 | <0.001 |
| Operation time>390 | 347 | 390 (298-490) | ref. | 1.285 | 0.114 | 1.539 | <0.001 | 1.355 | <0.001 | ref. | 0.771 | 0.021 | 1.207 | <0.001 | 1.224 | <0.001 |
| Intensive Care Unit (days)>3 | 257 | 3 (2-8) | ref. | -0.451 | 0.398 | -1.421 | <0.001 | 1.564 | <0.001 | ref. | -0.241 | 0.630 | 1.477 | <0001 | 1.690 | <0.001 |
| Hospital stay (days)>13 | 410 | 13 (9-21) | ref. | 0.337 | 0.492 | -0.383 | 0.218 | 0.460 | 0.036 | ref. | 0.465 | 0.104 | 0.569 | 0.019 | 0.384 | 0.077 |
| Readmission within 90 days | 70 | 0.209 | ref. | 0.932 | 0.114 | 0.105 | 0.810 | 0.500 | 0.070 | ref. | 1.289 | <0.001 | 1.007 | 0.003 | 0.501 | 0.051 |
|  |  |  |  |  |  |  |  |  |  |  |  |  |  |  |  |  |
| **Multiple Logistic regression (Log odds)** |  |  |  |  |  |  |  |  |  |  |  |  |  |  |  |  |
| Bile Leakage | 64 | 0,154 | ref. | 0.450 | 0.276 | -1.082 | 0.031 |  |  | ref. | 0.367 | 0.161 |  |  | -0.908 | 0.032 |
| Liver failure | 53 | 0,127 |  |  |  |  |  |  |  | ref. | 0.654 | 0.046 |  |  | 0.744 | 0.026 |
| Postoperative ascites | 94 | 0,226 | ref. | -0.162 | 0.788 | -1.381 | 0.052 | -0.985 | 0.135 | ref. |  |  | -0.559 | 0.061 |  |  |
| Postoperative bleeding | 43 | 0,103 |  |  |  |  |  |  |  | ref. | 2.042 | <0.001 | 1.32 | 0.026 | 0.923 | 0.056 |
| Pulmonary complications | 81 | 0,195 | ref. | 0.029 | 0.955 | -1.968 | 0.004 | -1.076 | 0.083 |  |  |  |  |  |  |  |
| Postoperative Infection | 104 | 0,25 |  |  |  |  |  |  |  | ref. |  |  | -0.253 | 0.299 |  |  |
| Cardiovascular complications | 27 | 0,064 |  |  |  |  |  |  |  | ref. | 1.692 | 0.038 | 2.194 | 0.004 |  |  |
| Cerebrovascular complications | 9 | 0,022 |  |  |  |  |  |  |  | ref. | 1.375 | 0.070 |  |  | 1.514 | 0.039 |
| Late biliary complications | 17 | 0,045 |  |  |  |  |  | 1.596 | 0.007 | ref. | 1.057 | 0.067 |  |  | 1.497 | 0.010 |
| Clavien ≥3a | 176 | 0,413 |  |  |  |  |  | 0.851 | 0.002 | ref. | 0.539 | 0.048 | 0.774 | 0.010 |  |  |
| Intraoperative transfusion | 218 | 0,549 |  |  |  |  |  |  |  | ref. | 1.391 | <0.001 | 1.103 | <0.001 | 1.196 | <0.001 |
| Intraoperative blood loss (ml) >718 | 328 | 750 (500-1800) |  |  |  |  |  |  |  | ref. | 1.569 | <0.001 | 0.727 | 0.070 | 1.338 | 0.001 |
| Operation time>390 | 347 | 390 (298-490) | ref. | 1.077 | 0.200 | -1.072 | 0.011 |  |  |  |  |  |  |  | 0.679 | 0.019 |
| Intensive Care Unit (days)>3 | 257 | 3 (2-8) |  |  |  |  |  | 0.731 | 0.055 | ref. | -0.947 | 0.018 | -0.037 | 0.930 | 0.960 | 0.020 |
| Hospital stay (days)>13 | 410 | 13 (9-21) |  |  |  |  |  |  |  |  |  |  |  |  |  |  |
| Readmission within 90 days | 70 | 0.209 |  |  |  |  |  |  |  | ref. | 0.981 | 0.002 |  |  | 0.953 | 0.004 |

**Supplementary Table 2.** Simple and multiple logistic regression analyses assessing the association between surgical approach and vascular control strategies with intraoperative and postoperative outcomes.

**Supplementary Table 3.** Perioperative characteristics and postoperative outcomes according to graft complications

| **Variable** | **Cohort (n = 260)*** | **Graft complications (n = 58)** | **p value** |
| --- | --- | --- | --- |
| **Type of hepatectomy** |  |  | 0.252 |
| Extended right hepatectomy | 53 (20.4%) | 7 (13.2%) |  |
| Right hepatectomy | 61 (23.5%) | 19 (31.1%) |  |
| Extended left hepatectomy | 40 (15.4%) | 7 (17.5%) |  |
| Left hepatectomy | 24 (9.2%) | 7 (29.2%) |  |
| Wedge resection | 29 (11.2%) | 7 (24.1%) |  |
| Other | 53 (20.4%) | 11 (20.8%) |  |
| **Need of regeneration technique** | 55 (21.2%) | 18 (32.7%) | 0.056 |
| **Liver perfusion** | 94 (36.2%) | 20 (21.3%) | 0.884 |
| **Type of occlusion** |  |  | 0.546 |
| Pringle | 72 (28.3%) | 13 (18.1%) |  |
| Partial | 39 (15.4%) | 8 (20.5%) |  |
| Total | 143 (56.3%) | 35 (24.5%) |  |
| **Time of vascular occlusion (min), median (IQR)** | 90 (63–120) | 100 (85–120) | 0.065 |
| **Use of extracorporeal bypass** | 93 (35.8%) | 23 (24.7%) | 0.41 |
| **Type of vascular repair** |  |  | 0.381 |
| Synthetic graft | 153 (58.8%) | 32 (19.3%) |  |
| Autologous graft | 57 (21.9%) | 16 (28.1%) |  |
| Xenograft | 21 (8.1%) | 3 (14.3%) |  |
| Allograft | 14 (5.4%) | 2 (14.3%) |  |
| Other | 15 (5.8%) | 1 (6.7%) |  |
| **Infiltrated vascular structure** |  |  |  |
| Inferior vena cava | 166 (63.8%) | 32 (19.3%) | 0.16 |
| Hepatic veins | 100 (38.5%) | 21 (21.0%) | 0.805 |
| Portal vein | 216 (40.6%) | 20 (34.5%) | 0.019 |
| Hepatic artery | 10 (3.8%) | 6 (60.0%) | 0.01 |
| **Approach type** |  |  | 0.685 |
| Ante-situ | 44 (16.9%) | 10 (22.7%) |  |
| Ex-situ | 24 (9.2%) | 7 (29.2%) |  |
| In-situ | 192 (73.8%) | 41 (21.4%) |  |
| **Intraoperative blood loss (ml), median (IQR)** | 900 (500–2500) | 1400 (525–2450) | 0.401 |
| **Intraoperative transfusion** | 135 (56.7%) | 31 (23.0%) | 0.492 |
| **Operation time (min), median (IQR)** | 390 (277.5–540) | 434.5 (302.2–597.5) | 0.057 |
| **Prophylactic postoperative anticoagulation** | 167 (68.7%) | 17 (10.2%) | 0.003 |
| **Therapeutic postoperative anticoagulation** | 111 (48.5%) | 40 (36.0%) | <0.001 |
| **Bile leakage** | 38 (14.6%) | 16 (42.1%) | 0.003 |
| **Liver failure** | 25 (9.6%) | 6 (24.0%) | 1 |
| **Postoperative bleeding** | 25 (9.6%) | 8 (32.0%) | 0.331 |
| **Postoperative infection** | 62 (23.8%) | 25 (40.3%) | <0.001 |
| **Clavien–Dindo ≥ IIIa** | 118 (45.4%) | 31 (26.3%) | 0.211 |
| **Clavien–Dindo ≥ IIIb** | 82 (31.5%) | 24 (29.3%) | 0.547 |
| **Clavien–Dindo V** | 28 (10.8%) | 8 (28.6%) | 1 |
| **Intensica care unit stay (days), median (IQR)** | 4 (2–8) | 5 (3–10) | 0.164 |
| **Hospital stay (days), median (IQR)** | 13 (9–24) | 15 (11–22) | 0.179 |
| **Readmission within 90 days** | 38 (15.3%) | 10 (26.3%) | 0.649 |

* Patients with data on graft complications. IQR: interquartile range.

**Supplementary table 4.** Comparison of surgical characteristics, perioperative management, and postoperative outcomes according to study period (1990–2010 vs. 2011–2024).

| **Variable** | **1990–2010 (n = 82)** | **2011–2024 (n = 450)** | **p** |
| --- | --- | --- | --- |
| **Approach type** |  |  |  |
| Ante-situ | 2 (2.4%) | 47 (10.4%) |  |
| Ex-situ | 10 (12.2%) | 19 (4.2%) | 0.002 |
| In-situ | 70 (85.4%) | 384 (85.3%) |  |
| **Infiltrated vascular structure** |  |  |  |
| Inferior vena cava | 68 (82.9%) | 187 (41.6%) | <0.001 |
| Hepatic veins | 19 (23.2%) | 125 (27.8%) | 0.466 |
| Portal vein | 10 (12.2%) | 206 (45.8%) | <0.001 |
| Hepatic artery | 1 (1.2%) | 30 (6.7%) | 0.068 |
| **Type of hepatectomy** |  |  |  |
| Extended right hepatectomy | 25 (30.5%) | 107 (23.8%) | 0.248 |
| Right hepatectomy | 18 (22%) | 109 (24.2%) |  |
| Extended left hepatectomy | 6 (7.3%) | 76 (16.9%) |  |
| Left hepatectomy | 9 (11%) | 51 (11.3%) |  |
| Wedge resection | 10 (12.2%) | 39 (8.7%) |  |
| Other | 14 (17.1%) | 68 (15.1%) |  |
| **Largest tumor size (mm), median (IQR)** | 80 (50–130) | 60 (34–89) | <0.001 |
| **Hypertrophy technique** | 2 (2.4%) | 106 (23.6%) | <0.001 |
| **Liver perfusion** | 22 (26.8%) | 100 (22.2%) | 0.441 |
| **Type of occlusion** |  |  |  |
| Pringle | 14 (17.5%) | 155 (37.7%) | <0.001 |
| Partial | 33 (41.2%) | 89 (21.7%) |  |
| Total | 33 (41.2%) | 167 (40.6%) |  |
| **Time of vascular occlusion (min), median (IQR)** | 45 (19.5–146.2) | 88 (45.8–110) | 0.117 |
| **Use of extracorporeal bypass** | 23 (28%) | 104 (23.1%) | 0.41 |
| **Type of vascular repair** |  |  |  |
| Primary suture | 24 (29.3%) | 212 (47.1%) | <0.001 |
| Synthetic graft | 49 (59.8%) | 130 (28.9%) |  |
| Autologous graft | 1 (1.2%) | 62 (13.8%) |  |
| Xenograft | 6 (7.3%) | 17 (3.8%) |  |
| Allograft | 1 (1.2%) | 14 (3.1%) |  |
| Other | 1 (1.2%) | 15 (3.3%) |  |
| **Intraoperative blood loss (ml), median (IQR)** | 1000 (750–1400) | 700 (425–1500) | 0.007 |
| **Intraoperative transfusion** | 43 (67.2%) | 211 (48.3%) | 0.003 |
| **Operation time (min), median (IQR)** | 420 (305–540) | 381.5 (300–484.2) | 0.313 |
| **Prophylactic anticoagulation** | 72 (96%) | 273 (63%) | <0.001 |
| **Therapeutic anticoagulation** | 6 (12%) | 208 (54.6%) | <0.001 |
| **Clavien ≥ IIIa** | 26 (31.7%) | 190 (42.2%) | 0.036 |
| **Clavien ≥ IIIb** | 20 (24.4%) | 132 (29.3%) | 0.097 |
| **Mortality** | 8 (9.8%) | 43 (9.6%) | 0.436 |
| **ICU stay (days), median (IQR)** | 3 (0–5) | 3 (1–8) | 1 |
| **Hospital stay (days), median (IQR)** | 14 (9–24) | 13 (9–21) | 0.351 |
| **Readmission within 90 days** | 8 (10.8%) | 80 (18.3%) | 0.734 |

ICU: intesnsive care unit; IQR: interquartile range
